# Pressure Ulcers and Nursing-Led Mobilization Protocols in ICU Patients: A Retrospective Observational Cohort Study

**DOI:** 10.3390/healthcare13141675

**Published:** 2025-07-11

**Authors:** Anna Korompeli, Eleni Karakike, Petros Galanis, Pavlos Myrianthefs

**Affiliations:** Department of Health Science, Faculty of Nursing, National and Kapodistrian University of Athens, 11527 Athens, Greece; elkarakike@nurs.uoa.gr (E.K.); pegalan@nurs.uoa.gr (P.G.); pmiriant@nurs.uoa.gr (P.M.)

**Keywords:** pressure ulcer, ICU, nursing care, mobilization, SOFA score, pressure injury prevention

## Abstract

**Background:** Pressure ulcers (PUs) remain a prevalent complication in intensive care unit (ICU) settings, especially among immobilized patients. The impact of structured, nursing-led mobilization protocols on PU prevention and recovery remains underexplored. **Objective:** To evaluate the impact of nursing-led mobilization protocols on the incidence and progression of PUs in critically ill patients. **Methods:** In this retrospective observational cohort study, 188 ICU patients were admitted during one of two consecutive periods of care: conventional care (6-hourly repositioning) and an advanced nursing-led protocol (3-hourly repositioning with support surfaces and specialized nurse training), which replaced conventional care as standard in our institution. The primary outcome included new PU development for patients with no pre-existing ulcers or worsening/non-progression of pre-existing ulcers at discharge; ICU mortality was evaluated as a secondary outcome. **Results:** Among patients without pre-existing ulcers (n = 155), new PU incidence did not significantly differ between groups, even after adjusting for SOFA score (OR 0.40, 95% CI: 0.05 TO 3.17; *p* = 0.374). However, in patients with pre-existing ulcers (n = 33), the advanced care group showed improvement (53.3% versus 0% in the conventional group, OR 0.07, 95% CI: 0.01–0.64; *p* = 0.012); this effect was independent of initial SOFA score. Mortality was associated with the SOFA score, but not with the type of care. **Conclusions:** While advanced nursing-led mobilization did not reduce PU incidence, it significantly improved existing ulcer outcomes. Findings support the integration of structured protocols for high-risk ICU patients, especially those with existing ulcers.

## 1. Introduction

Pressure ulcers (PUs), also known as pressure injuries or bedsores, remain a prevalent and serious complication in Intensive Care Units (ICUs), with incidence rates ranging from 10% to over 30% depending on patient condition and care practices [1,2,3]. These injuries occur from prolonged pressure on the skin, leading to ischemia, tissue breakdown, and increased morbidity. In critically ill populations, the consequences of pressure ulcers beyond discomfort contribute to extended hospital stays, increased healthcare costs, and elevated in-hospital mortality.

Given the high vulnerability of ICU patients—many of whom are sedated, ventilated, or otherwise immobilized—early and consistent mobilization has emerged as a key strategy for PU prevention. Evidence suggests that mobilization enhances tissue perfusion, reduces the risk of skin breakdown, and supports overall physiological recovery [4,5]. However, ICU settings present unique barriers to patient movement, including hemodynamic instability, sedation, and limited staffing, which can hinder effective mobilization efforts.

Nurses play a central role in implementing mobilization protocols, often serving as the primary drivers of early movement and repositioning routines. Despite growing recognition of nursing-led mobilization as a critical preventive strategy, clinical practices vary widely, particularly in terms of protocol adherence, timing, and intervention type. While conventional care often involves intermittent repositioning and passive mobilization, advanced nursing-led protocols typically integrate structured schedules, interdisciplinary collaboration, specialized support surfaces, and enhanced staff education [6].

This study aimed to assess the impact of an advanced nursing-led protocol of mobilization, as compared to conventional care, on the incidence and recovery of pressure ulcers and on in-hospital mortality among ICU patients. Our findings seek to inform evidence-based guidelines and support the broader implementation of nursing-driven prevention protocols in critical care environments.

## 2. Materials and Methods

### 2.1. Study Design

This is a retrospective observational cohort study, where patients were exposed to one of two sequential types of care regarding PU; one conventional and one including advanced and proactive screening and mobilization. As part of the institutional quality improvement program, our policy regarding patient mobilization and PU care was intensified (29/11/2023, PROTOCOL NUMBER 1067). Patients admitted during a period of 6 months before and those admitted 6 months after this policy change were compared. Thus, all care exposures occurred within the framework of routine clinical practice, without investigator-driven modifications, conferring a non-interventional design.

### 2.2. Setting

This study was conducted between January 2024 and February 2025 in the Academic General Intensive Care Unit (ICU) of a tertiary-care general hospital in Athens, Greece. Data were retrospectively extracted from medical records. Follow-up ended at discharge from the ICU or at 90 days after admission, whichever occurred first.

### 2.3. Participants

The study included all consecutive adult ICU patients (aged ≥18 years) with a minimum ICU stay of 24 h, admitted between January 2024 and February 2025. No exclusion criterion was applied. Patients with pre-existing pressure ulcers at admission were included and analyzed separately from those without PUs at baseline, to enable full evaluation of new ulcer development during hospitalization.

Patients were divided into two groups, based on the type of care that was applicable on the date of their admission to the ICU:

- The conventional care group received standard ICU care, including repositioning approximately every 6 h, as per routine practices, without structured staff education or interdisciplinary mobilization strategies. Conventional care was delivered from January to August 2024.
- The advanced nursing-led mobilization care was delivered from September 2024 to February 2025 and emphasized coordinated nursing-led care; a Pressure Ulcer Prevention Protocol (PUPP) [7], implemented as part of an advanced nursing care model. Also, repositioning was conducted every three hours, with documented collaboration between nursing and medical teams. Nurses received targeted training in pressure injury risk assessment and skin integrity management. Specialized pressure-relieving support surfaces and protective foam dressings were used to minimize skin breakdown. Each patient had an individualized mobilization plan tailored to their tolerance and clinical stability, incorporating both passive and active exercises. The protocol also included regular skin inspection routines, the use of barrier creams, and daily monitoring of skin condition. Evaluation of PU progression included targeted assessment of skin integrity over bony prominences, which are critical early indicators of ulcer development. This was emphasized during staff training and incorporated into ongoing patient monitoring protocols. This approach was designed to address known barriers to mobilization in ICU settings, such as hemodynamic instability, sedation, medical equipment constraints, and limited staffing [6,8,9,10]. Structured massage over bony areas was not incorporated into our institutional protocol, due to concerns regarding tissue fragility in critically ill patients.
  For both groups, nutritional assessment was performed as part of the standard medical practice, and patients received high-protein diets and hydration plans to support tissue repair and healing. Also, over time, PU incidence and progress are systematically and prospectively recorded by the nursing team in a separate individual document remaining in the patient file.

### 2.4. Data Collection and Outcome Measures

The following data were collected from the medical file:

- Demographics (age, sex).
- Reason for admission to the ICU (medical or surgical).
- Risk stratification was carried out using the Cubbin & Jackson pressure injury risk scale [2].
- Clinical variables of severity at ICU admission (the acute pathophysiology and chronic health evaluation -APACHE II and sequential organ failure assessment-SOFA scores), and the use of organ support, mechanical ventilation, vasopressor use and dose, and Renal Replacement Therapy [RRT].
- Pre-existing pressure ulcers

Outcomes:

The primary outcome assessed was (i) development of (new) pressure ulcers during hospitalization, for patients without pre-existing ulcers, and (ii) worsening or non-improvement for those with pre-existing pressure ulcers. Worsening and non-improvement were defined by progression or no change in the Push score [11] at discharge from the ICU.

ICU mortality was evaluated as a secondary outcome. Mortality analysis was based on ICU outcomes within 90 days of admission. Patients who were discharged alive before 90 days were considered survivors. For patients still hospitalized in the ICU at 90 days, mortality status was recorded up to that point.

### 2.5. Study Size

As this was a retrospective analysis of data collected over a defined timeframe, no formal sample size calculation was conducted.

### 2.6. Statistical Analysis

Categorical variables were presented as numbers and percentages. Continuous variables were presented with their mean and standard deviation (SD). We considered the clinical characteristics of patients as independent variables. We considered ulcer occurrence, ulcer worsening/non-progression, and death during ICU hospitalization as the dependent variables. We first performed univariate and forward stepwise multivariable binary logistic regression analyses to examine the net and independent impact of independent variables on ulcer occurrence and death. We did not assess ulcer worsening/non-progression at this stage, since we considered that it is affected by the same risk factors as PU occurrence. Since APACHE score and SOFA score were highly correlated (Pearson’s correlation coefficient = 0.73, *p*-value < 0.001), as well as Cubbin & Jacksson and SOFA (Pearson’s correlation coefficient = 0.43, *p*-value < 0.001) we included only one (SOFA score) of them in the multivariable model to avoid multicollinearity issues. We then assessed the effect of the advanced care protocol on ulcer occurrence, ulcer worsening/non-progression, and death. We present unadjusted and adjusted odds ratios (ORs), 95% confidence intervals (CIs), and *p*-values, based on the factors identified as independently associated with outcomes in the previously constructed multivariable model. We confirmed the findings by Kaplan–Meier survival and Cox regression analyses to illustrate the effect of the advanced nursing-led care on outcomes and present unadjusted hazard ratios (HR), with respective 95% CI for the time to ulcer occurrence and death. All two-tailed *p*-values less than 0.05 were considered statistically significant. We used the IBM SPSS 28.0 (IBM Corp. Released 2021. IBM SPSS Statistics for Windows, Version 28.0. Armonk, NY, USA: IBM Corp) for the analysis.

### 2.7. Ethics

The project (procedures of care and prospective data recording) was part of a standing IRB approval under protocol No. 1067/November 29, 2023. For publication purposes, an additional, updated IRB approval was requested to include the exact characteristics of this analysis, which was granted on 25 June 2025 under protocol No. 520. All patient data were anonymized, meaning all identifying information was irreversibly removed, in accordance with ethical standards. Informed consent was waived as either care modality was considered the standard of care for the respective periods where it was applied.

## 3. Results

### 3.1. Clinical Characteristics

Between January 2024 and February 2025, 188 consecutive patients were included, 77 (41.0%) in the advanced and 111 (59.0%) in the conventional care group. Clinical characteristics on the first day of admission are shown in Table 1. Overall, 93 females (49.5%) and 95 males (50.5%) were included, with a mean age of 69.4 years (SD, 14.1), a mean SOFA score of 8.4 (SD, 3.9), and a mean APACHE II of 19.9 (SD, 8.1). All (100%) patients were intubated, 148 (78.7%) received noradrenaline, and 55 (29.3%) received RRT. Mean Cubbin & Jacksson score was 24.4 (SD, 3.9). No significant differences were noted between the groups of care (Table 1).

### 3.2. Primary Outcome

#### Incidence of Pressure Ulcers During Hospitalization, Among Patients Without Pre-Existing Ulcers

**Figure 1:**
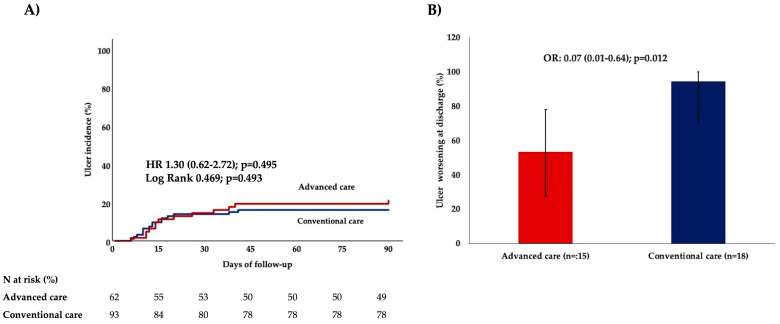
Effect of an advanced nursing-led mobilization protocol on ulcer outcomes (**A**) ulcer incidence among patients with no pre-existing ulcers (n = 155) and (**B**) ulcer worsening or non-progression among those with pre-existing ulcers at admission (n = 33). HR and OR, with respective 95% confidence intervals (in brackets) and *p*-values, are provided by Cox-regression (panel (**A**)) and Logistic regression analyses (panel (**B**)), respectively. The Log-rank test comparing the overtime distribution of ulcer incidence by Kaplan–Meier analysis is also provided (panel (**A**)). Abbreviations: HR: hazard ratio, OR: odds ratio.

A total of 155 patients were admitted without pre-existing ulcers, and 28 (18.6%) of them developed new ulcers during hospitalization. Incidence was similar in the advanced Care (13 out of 62; 16.1%) and the conventional Care groups (15 out of 93; 21%), with an OR of 1.38 (95% CI: 0.605–3.145; *p* = 0.524) (Figure 1A).

We performed logistic regression analysis to identify factors associated with ulcer occurrence in the entire cohort. Multivariable analysis identified only the SOFA score as an independent parameter associated with ulcer occurrence (Table 2).

We then adjusted our assessment of the advanced care protocol for the baseline SOFA score. Even after adjustment, the advanced care protocol was not associated with decreased ulcer incidence (adjusted OR 0.40, 95% CI: 0.05 to 3.17; *p*-value = 0.374).

### 3.3. Worsening of Pre-Existing Pressure Ulcers

Among the 33 patients with pre-existing ulcers, worsening or no progression was observed in 26 (78.8%) of cases. Advanced care was associated with improvement in 8 of 15 cases (53.3%) while none of the 18 (0%) patients receiving conventional care showed improvement, yielding an OR for advanced care of 0.07 (95% CI: 0.01–0.64); *p* = 0.012, using the Firth correction. The advanced nursing-led care was associated with improvement, even after adjustment for SOFA score (adjusted OR 0.07, 95% CI: 0.01–0.64; *p* = 0.019). (Figure 1B).

### 3.4. ICU Mortality

**Figure 2:**
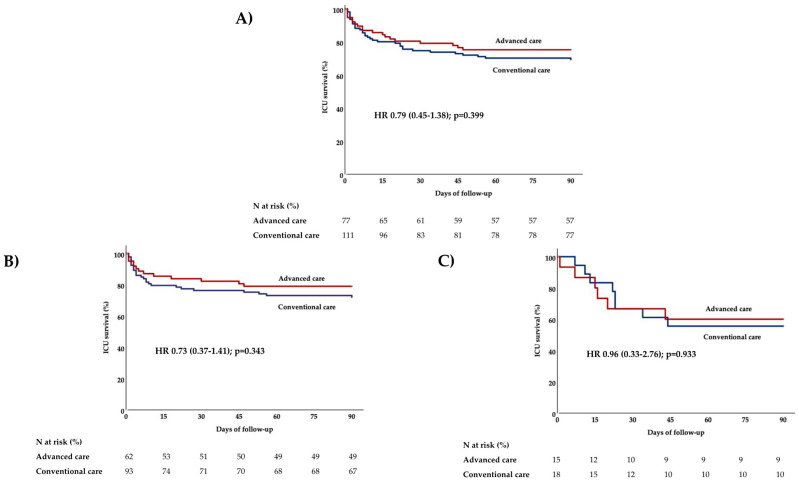
Effect of advanced nursing-led mobilization protocol on 90-day ICU mortality (**A**) in the entire study population (n = 188), (**B**) among patients without pre-existing ulcers (n = 155), and (**C**) among those with pre-existing ulcers at admission (n = 33). *p*-values are provided by Cox-regression analysis. Abbreviations: HR: hazard ratio, ICU: intensive care unit.

By the end of ICU stay or by 90 days, whichever was earlier, 53 (28.2%) patients had died. The advanced care protocol was not associated with mortality (Figure 2), in either subgroup; among patients without pre-existing ulcers, 13/62 (21%) died in the advanced care group, compared to 26/93 (28%) in the conventional care group, OR 0.68 (95% CI 0.32–1.46; *p* = 0.351). Among patients with pre-existing ulcers (n = 33), the advanced care group had a mortality rate of 40.0% (6 deaths out of 15), while the conventional care group had a rate of 44.4% (8 deaths out of 18). Again, the difference was not statistically significant (OR 0.83, 95% CI 0.21–3.35; *p* = 1.000).

Logistic regression models with deaths during ICU hospitalization as the dependent variable in the overall cohort are shown in Table 3. After elimination of confounders, the only variable independently associated with mortality was the SOFA score.

After adjustment for baseline SOFA score, the advanced care protocol remained insignificantly associated with ICU mortality, both in the entire cohort (adjusted OR 0.76; 95% CI 0.35–1.63; *p* = 0.478) and in the subgroups of patients without (adjusted OR 0.73; 95% CI 0.29–1.83; *p* = 0.502) and with pre-existing ulcers (adjusted OR 0.82; 95% CI 0.20–3.36; *p* = 0.777).

Nevertheless, in the subgroup of patients with pre-existing ulcers, ICU mortality was significantly associated with ulcer worsening (7/7–100% of patients with no worsening survived compared with 12/26, 46.2% of patients surviving with worsening ulcers; *p* = 0.013).

## 4. Discussion

This study examined the effect of nursing-led mobilization protocols on pressure ulcer incidence among ICU patients, comparing outcomes between conventional care and an advanced, multifaceted nursing-led care. The findings underscore the significant role of physiological severity, as indexed by the SOFA score, in predicting adverse outcomes in critically ill patients.

### 4.1. Pressure Ulcer Incidence and Progression

A total of 155 patients were admitted without pre-existing ulcers, and 28 (18.6%) of them developed new ulcers during hospitalization, consistent with the literature [3,12]. While the incidence of new PUs did not differ significantly between the two groups, advanced care protocols showed a beneficial impact on the progression of pre-existing ulcers; this impact was independent of the initial severity of illness. This result suggests that the presence of a pre-existing ulcer at ICU admission should trigger a heightened clinical response from nursing staff, leading to more proactive interventions aimed at preventing ulcer deterioration. This can be explained by several factors related to the nature of ulcers and their impact on patient care. A pre-existing ulcer indicates a patient is already experiencing a health issue and may be at higher risk for complications. This can heighten the nurse’s awareness and concern for the patient’s well-being. Moreover, the presence of an ulcer often necessitates specific care measures, and nurses are likely to be more proactive in implementing these measures to prevent further deterioration or infection. Nurses are trained to prevent the development of pressure ulcers, and the presence of an existing one can serve as a strong reminder to assess the patient’s risk factors and implement preventative strategies [13,14]. Notably, improvement was observed in over half of the patients receiving advanced care, compared to none in the conventional care group. This suggests that while prevention may require broader system-level support, structured protocols are more effective at mitigating progression in already vulnerable patients [12].

Also, advanced care was not more effective than conventional care in reducing new ulcer formation. An individual stratification of risk should be undertaken to avoid unnecessary resource allocation and healthcare worker mobilization for patients without an ulcer at admission. Moreover, this may reflect challenges in implementation fidelity, variation in staff adherence, or patient-specific factors such as hemodynamic instability and sedation, common barriers in ICU mobilization efforts, and is applicable to both strategies. Previous multicenter studies support the notion that the efficacy of mobilization protocols depends heavily on consistent application and interdisciplinary collaboration.

We show that the SOFA (Sequential Organ Failure Assessment) score, a measure of organ dysfunction, is an important factor associated with the incidence of pressure ulcers (PUs) in critically ill patients. This is consistent with prior studies, potentially due to factors like reduced mobility, impaired tissue oxygenation, and weakened immune function [15].

### 4.2. Mortality and Predictive Indicators

Mortality analysis paralleled PU outcomes: the SOFA score retained its predictive power in multivariable models, underscoring physiological severity as a key determinant of ICU survival [15].

Despite enhancements in nursing care, advanced care protocols were not associated with improved survival. This may further highlight that clinical outcomes are not solely dependent on protocol design, but also on individualized patient acuity and system readiness [16]. However, ulcer worsening was associated with higher mortality among patients with pre-existing ulcers, possibly indicating a mediator effect of advanced protocols on final outcome [15].

### 4.3. Interpretation and Clinical Implications

Overall, these findings emphasize the need for early, structured mobilization as a core component of ICU nursing care. While our advanced protocol incorporated evidence-based elements—including risk stratification, enhanced materials, and staff education—the absence of statistically significant differences between groups highlights the complex interplay between patient acuity and staffing resources.

Our study supports the importance of stratifying patients early and guiding individualized prevention strategies. The most vulnerable patients with pre-existing ulcers might benefit the most from advanced nursing-led care, while standard mobilization protocols should be common practice for all other patients. Nursing care teams should be empowered with ongoing training, multidisciplinary coordination, and supportive equipment to translate prevention protocols into consistent bedside practice.

### 4.4. Limitations and Strengths

This study has several limitations that should be acknowledged. Firstly, it was conducted in a single tertiary care ICU, which may limit the generalizability of the findings to other settings or patient populations. Secondly, the retrospective nature of this analysis of prospectively collected data did not allow for several factors to be systematically assessed in the present work; non-recording of the precise timing of pressure ulcer progression or improvement, limits interpretation of temporal trends; similarly, lack of capturing the timing of adherence to mobilization protocols may have introduced bias against the advanced protocol, due to delays of application among the most critically ill patients (with hemodynamic instability or continuous renal replacement therapy); comorbidity index and nutritional status was only recorded as part of the APACHE II and the Cubbin-Jackson scale, respectively, and not as separate variables, limiting accounting for these factors in the final outcome. We also acknowledge that a more detailed analysis of underlying conditions, such as sepsis and respiratory status, could further inform outcomes and should be considered in future prospective studies. Finally, the observational design based on two consecutive periods of care, without randomization, includes the potential for residual confounding, even though the two groups were found to be balanced in their baseline characteristics. Despite its limitations, this study offers several important contributions. It compares the real-world implementation of conventional versus advanced nursing protocols, identifies a new at-risk patient population (those with pre-existing ulcers), and emphasizes the central role that severity of illness plays in PU risk prediction. To address these issues, future prospective multicenter studies are necessary to validate the findings and assess the optimal mobilization protocol intensity on patient outcomes.

## 5. Conclusions

In conclusion, physiological severity remains the most significant factor associated with pressure ulcer development and mortality in ICU patients. While conventional care should be delivered to avoid new ulcer occurrence, more advanced nursing-led mobilization protocols should be applied for patients with existing ulcers, with a possible intermediate effect on mortality. Enhancing adherence to evidence-based mobilization strategies is essential for translating protocol efficacy into improved patient outcomes. Future research should focus on multicenter validation and the development of standardized, feasible mobilization protocols tailored to ICU patient populations.

## Figures and Tables

**Table 1:** Clinical characteristics of patients upon admission to the Intensive Care Unit.

Characteristics	Total N = 188	Advanced Care (n = 77)	Conventional Care (n = 111)	*p* Value
Gender, n (%)				0.182
Females	93 (49.5)	43 (55.8)	50 (45.0)	
Males	95 (50.5)	34 (44.2)	61 (55.0)	
Age, mean (standard deviation)	69.4 (14.1)	69.7 (13.9)	69.2 (14.3)	0.958
RRT, n (%)				0.103
No	133 (70.7)	49 (63.6%)	84 (75.7%)	
Yes	55 (29.3)	28 (36.4%)	27 (24.3%)	
Mechanical ventilation, n (%)	188 (100)	77 (100)	111 (100)	1.00
Noradrenaline support, n (%)				0.590
No	40 (21.3)	18 (23.4)	22 (19.8)	
Yes	148 (78.7)	59 (76.6)	89 (80.2)	
Noradrenaline dose (γ/kg/min), mean (sd)	11.4 (11.4)	12.5 (11.8)	10.6 (11.0)	0.294
APACHE II score, mean (standard deviation)	19.9 (8.1)	20.2 (8.0)	19.7 (8.1)	0.702
SOFA score, mean (standard deviation)	8.4 (3.9)	8.3 (4.2)	8.5 (3.6)	0.619
CUBBIN & JACKSSON scale, (mean, sd)	24.4 (3.9)	24.4 (3.5)	23.8 (3.7)	0.423
Reasons for ICU admission, n (%)				0.186
Medical	122	46 (59.7)	76 (68.5)	
Surgical (Postoperative patients)	66	31 (40.3)	35 (31.5)	
Pre-existing pressure ulcer, n (%)				0.565
No	155 (82.4)	62 (80.5)	93 (83.8)	
Yes	33 (17.6)	15 (19.5)	18 (16.2)	

Abbreviations: APACHE: acute pathophysiology and chronic health evaluation; RRT: renal replacement therapy; SOFA: sequential organ failure assessment.

**Table 2:** Logistic regression models were used with ulcer occurrence during hospitalization as the dependent variable in the entire cohort (N = 188).

Variable	Univariate Models			Multivariable Models		
	Unadjusted OR	95% CI for OR	*p*-Value	Adjusted OR	95% CI for OR	*p*-Value
Male sex	0.73	0.35–1.54	0.410	0.56	0.25–1.27	0.162
Age	1.00	0.98–1.03	0.809	1.07	0.98–1.04	0.674
RRT	3.05	1.42–6.56	0.004	1.83	0.80–4.20	0.152
Vasopressor use	10.85	1.44–81.97	0.021	0.756	0.976–58.5	0.053
SOFA score	1.18	1.07–1.31	0.001	1.13	1.01–1.26	0.043
Surgical reason for admission	0.198	0.07–0.51	0.004	0.31	0.10–1.02	0.054

Abbreviations: CI: confidence interval; OR: odds ratio; RRT: renal replacement therapy; SOFA: sequential organ failure assessment.

**Table 3:** Logistic regression models with ICU mortality by 90 days as the dependent variable in the entire cohort (N = 188).

Variable	Univariate Models			Multivariable Models		
	Unadjusted OR	95% CI for OR	*p*-Value	Adjusted OR	95% CI for OR	*p*-Value
Male sex	1.56	0.82–2.97	0.173	1.24	0.58–2.66	0.578
Age	1.03	0.99–1.05	0.054	1.03	0.99–1.06	0.085
RRT	2.78	1.42–5.44	0.003	1.18	0.51–2.78	0.705
Vasopressor use	6.29	1.85–21.42	0.003	3.72	0.43–32.07	0.232
SOFA score	1.39	1.24–1.56	<0.001	1.40	1.24–1.57	<0.001
Pre-existing ulcer	2.19	1.01–4.78	0.049	1.77	0.73–4.26	0.247
Surgical reason for admission	0.44	0.12–0.91	0.027	1.79	0.68–4.75	0.241

Abbreviations: CI: confidence interval; OR: odds ratio; RRT: renal replacement therapy; SOFA: sequential organ failure assessment.

## Data Availability

De-identified patient data may be available upon request to the corresponding author after careful consideration.
